# Evaluation of Cervical Cancer Screening Uptake and Adherence Among Women Without Human Papillomavirus Vaccination in the US

**DOI:** 10.1001/jamanetworkopen.2021.31129

**Published:** 2021-10-26

**Authors:** Kalyani Sonawane, Ryan Suk, Elizabeth Y. Chiao, Kathleen M. Schmeler, Jane Montealegre, Maria E. Fernandez, Ashish A. Deshmukh

**Affiliations:** 1 Center for Health Services Research, Department of Management, Policy & Community Health, UTHealth School of Public Health, Houston, Texas; 2 Center for Healthcare Data, Department of Management, Policy & Community Health, UTHealth School of Public Health, Houston, Texas; 3 Department of Epidemiology, Division of Cancer Prevention and Population Sciences, MD Anderson Cancer Center, Houston, Texas; 4 Department of Gynecologic Oncology and Reproductive Medicine, MD Anderson Cancer Center, Houston, Texas; 5 Department of Pediatrics, Baylor College of Medicine, Houston, Texas; 6 Center for Health Promotion and Prevention Research, UTHealth School of Public Health, Houston, Texas

## Abstract

This cross-sectional study compares uptake rates of cervical cancer screening and adherence among US women who were vaccinated for human papillomavirus vs those who were not vaccinated.

## Introduction

Human papillomavirus (HPV) vaccination and cervical cancer screening are the cornerstone interventions to achieve cervical cancer elimination.^1^ Given that 46% of adolescent and 65% of young women are not up to date on the HPV vaccination series in the US, screening is an indispensable measure for cervical cancer elimination.^1,2^ However, a sharp decline in cervical cancer screening rates among young women has occurred in recent years.^3^ Unvaccinated women who have not had a screening may remain susceptible to cervical cancer. Herein, we examined cervical cancer screening uptake and adherence among women in the US in 2019 stratified by HPV vaccination status.

## Methods

This cross-sectional study analyzed National Health Interview Survey data from 2019. The survey participants self-reported sociodemographic information; however, race and ethnicity information was selected according to defined categories in the National Health Interview Survey, including Hispanic, non-Hispanic Asian, non-Hispanic Black, non-Hispanic White, and other (non-Hispanic American Indian/American Natives only, non-Hispanic American Indian/American Natives and any other group, other single race and multiple races). The study population included women who were ever eligible to receive the HPV vaccine (ie, those aged 9-26 years between the vaccine’s licensure from 2006 to 2019) and were eligible for cervical cancer screening on the basis of age (ie, those aged 21-39 years for the purposes of this study). We excluded women who underwent a hysterectomy and those with missing information on HPV vaccination and cervical cancer screening. The outcomes of interest were (1) ever screened for cervical cancer and (2) up to date on screening on the basis of the US Preventive Services Task Force recommendations.^4^ A survey design–adjusted Wald *F* test was used to compare cervical cancer screening uptake and up-to-date status stratified by HPV vaccination status. We estimated the odds ratio for screening uptake and up-to-date status among unvaccinated women using multivariable logistic regression models. Statistical significance was tested at 2-sided *P* = .05. All analyses were performed with SAS software, version 9.4 (SAS Institute) using SAS PROC SURVEY procedures and adjusting for the complex survey design and sampling weights. The institutional review board of the University of Texas Health Science Center deemed this study exempt from review and waived the requirement for informed consent owing to the use of publicly available data. The study followed the Strengthening the Reporting of Observational Studies in Epidemiology (STROBE) reporting guideline.

## Results

The National Health Interview Survey from 2019 included data for 1872 women aged 21 to 29 years (mean [SE] age, 25.1 [0.1] years) who were predominately White individuals (54.1%) with some college education (35.8%), private insurance (62.4%), and urban status (89.3%) living in the South (40.1%). Of these women, 24.6% never underwent cervical cancer screening, and 29.1% did not adhere to screening recommendations. Among 2637 women aged 31 to 39 years (mean [SE] age, 34.5 [.01] years) (most were White individuals [54.2%] with some college education [29.5%], private insurance [65.2%], and urban status [87.4%] who were living in the South [37.8%]), 11.5% never underwent cervical cancer screening, and 17.5% did not adhere to screening recommendations.

Among women aged 21 to 29 years who were not vaccinated for HPV, a higher proportion reported never receiving cervical cancer screening compared with vaccinated women (32.2% vs 17.9%; *P* < .001) (Table 1). Similarly, a greater proportion of unvaccinated women were not up to date on cervical cancer screening recommendations compared with their vaccinated counterparts (37.4% vs 21.6%; *P* < .001). Findings were similar for women aged 30 to 39 years; a higher proportion of unvaccinated women vs vaccinated women were never screened (13.5% vs 5.0%; *P* < .001) and did not adhere to screening recommendations (19.9% vs 9.3%; *P* < .001).

**Table 1.** Cervical Cancer Screening Uptake and Up-to-Date Status by Human Papillomavirus Vaccination Status Among US Women in 2019^a^

	Aged 21-29 y (n = 1872)		*P* value	Aged 30-39 y (n = 2637)		*P* value
	Vaccinated (received ≥1 dose)	Unvaccinated		Vaccinated (received ≥1 dose)	Unvaccinated	
**Never screened**						
Proportion of women, % (95% CI)	17.9 (14.8-21.0)	32.2 (28.3-36.1)	<.001	5.0 (2.9-7.0)	13.5 (11.5-15.5)	<.001
Unweighted No./total No. of women	138/950	233/790		31/577	221/1883	
Weighted No./total No. of women	1 722 553/9 629 751	2 630 360/8 179 391		214 484/4 324 239	1 968 956/14 615 577	
**Not up to date according to screening recommendations^b^**						
Proportion of women, % (95% CI)	21.6 (18.2-25.0)	37.4 (33.3-41.5)	<.001	9.3 (6.4-12.1)	19.9 (17.6-22.3)	<.001
Unweighted No./total No. of women	175/948	271/786		56/573	332/1852	
Weighted No./total No. of women	2 075 773/9 592 209	3 034 917/8 121 639		398 371/4 306 412	2 864 694/14 361 041	

^a^ Based on nonmissing National Health Interview Survey data on cervical cancer screening behavior and human papillomavirus vaccination, cervical cancer screening data were missing for 38 patients (15 patients aged 21 to 29 y; 23 patients aged 30 to 39 y), screening adherence data were missing for 85 patients (22 patients aged 21 to 29 y; 63 patients aged 30 to 39 y), and human papillomavirus vaccination information was missing for 281 patients (121 patients aged 21 to 29 y; 160 patients aged 30 to 39 y).

^b^ Up-to-date status for women aged 21 to 29 y was established by considering whether they had received a Papanicolaou test within the past 3 years. Up-to-date screening for women aged 30 to 39 y was either having a Papanicolaou test within the past 3 years or having a Papanicolaou test with an HPV cotest within the past 5 years.

Among women aged 21 to 29 years who were not vaccinated for HPV, the likelihood of never receiving cervical cancer screening was higher for non-Hispanic Asian women (adjusted odds ratio [AOR], 2.07; 95% CI, 1.03-4.16) and women with an educational level up to a high school diploma (AOR, 2.33; 95% CI, 1.07-5.12) (Table 2). Among women aged 30 to 39 years, the likelihood of never receiving a cervical cancer screening was higher for Hispanic (AOR, 2.77; 95% CI, 1.71-4.51), non-Hispanic Asian (AOR, 3.03; 95% CI, 1.70-5.40), and non-Hispanic Black women (AOR, 2.25; 95% CI, 1.37-3.69) as well as those with educational attainment up to a high school diploma (AOR, 1.89; 95% CI, 1.11-3.21) and those who were uninsured (AOR, 2.46; 95% CI, 1.56-3.88). Findings were similar for women who were not up to date on screening recommendations.

**Table 2.** Proportions and Adjusted Odds Ratios for Having Never Been Screened or Not Adhering to Cervical Cancer Screening Guideline Among Unvaccinated US Women

Factor	Aged 21-29 y (n = 790)				Aged 30-39 y (n = 1883)			
	Never screened		Not up to date		Never screened		Not up to date	
	Proportion, % (95% CI)	AOR (95% CI)	Proportion, % (95% CI)	AOR (95% CI)	Proportion, % (95% CI)	AOR (95% CI)	Proportion, % (95% CI)	AOR (95% CI)
Race and ethnicity								
Hispanic	32.3 (26.8-43.7)	1.07 (0.66-1.75)	43.0 (34.6-51.4)	1.09 (0.69-1.72)	25.2 (19.5-30.9)	2.77 (1.71-4.51)^a^	31.0 (25.1-36.8)	1.66 (1.11-2.49)^a^
Non-Hispanic								
Asian	46.5 (32.0-60.9)	2.07 (1.03-4.16)^a^	47.7 (33.3-62.1)	1.72 (0.87-3.42)	17.6 (11.3-23.9)	3.03 (1.70-5.40)^a^	21.3 (14.5-28.1)	1.92 (1.12-3.31)^a^
Black	28.3 (17.1-39.4)	0.86 (0.45-1.64)	29.5 (18.2-40.8)	0.66 (0.34-1.28)	16.8 (11.4-22.3)	2.25 (1.37-3.69)^a^	20.3 (14.3-26.3)	1.16 (0.73-1.84)
White	30.1 (24.7-35.4)	[Reference]	35.6 (29.7-41.4)	[Reference]	7.4 (5.4-9.4)	[Reference]	15.4 (12.6-18.3)	[Reference]
Other^b^	33.3 (10.2-56.4)	1.25 (0.44-3.51)	44.8 (19.6-69.9)	1.57 (0.56-4.40)	3.5 (0.0-8.0)	0.33 (0.09-1.27)	11.1 (0.0-22.7)	0.45 (0.12-1.68)
Educational level								
Graduate degree	22.8 (11.0-34.7)	[Reference]	28.8 (15.4-42.3)	[Reference]	9.0 (5.6-12.4)	[Reference]	11.2 (7.2-15.3)	[Reference]
Bachelor’s degree	29.0 (21.8-36.2)	1.56 (0.71-3.40)	32.1 (24.6-39.5)	1.26 (0.59-2.70)	7.1 (4.4-9.6)	0.77 (0.44-1.36)	11.2 (8.0-14.3)	0.95 (0.57-1.57)
Some college	29.8 (22.5-37.1)	1.70 (0.77-3.77)	33.4 (25.9-40.9)	1.35 (0.63-2.89)	8.9 (5.7-12.1)	0.85 (0.48-1.50)	16.9 (12.9-20.8)	1.22 (0.73-2.02)
Up to high school diploma	36.7 (30.2-43.1)	2.33 (1.07-5.12)^a^	44.4 (37.5-52.3)	2.03 (0.94-4.38)	24.0 (19.5-28.6)	1.89 (1.11-3.21)^a^	32.9 (28.0-37.9)	2.04 (1.24-3.35)^a^
Urban or rural status								
Urban	32.6 (28.4-36.7)	[Reference]	37.7 (33.3-42.1)	[Reference]	13.2 (11.1-15.4)	[Reference]	18.6 (16.2-21.0)	[Reference]
Rural	28.7 (17.8-39.7)	0.82 (0.46-1.47)	34.4 (23.5-45.4)	0.79 (0.46-1.37)	15.0 (8.8-21.2)	1.52 (0.90-2.58)	29.4 (21.8-37.0)	2.03 (1.34-3.07)^a^
Insurance status								
Private	30.8 (25.5-36.1)	[Reference]	33.6 (28.2-38.9)	[Reference]	8.5 (6.5-10.4)	[Reference]	12.0 (9.7-14.2)	[Reference]
Public	33.4 (24.9-41.9)	1.03 (0.61-1.73)	40.8 (32.0-49.5)	1.26 (0.76-2.09)	18.4 (13.2-23.8)	1.60 (0.97-2.67)	28.3 (22.2-34.4)	2.06 (1.34-3.16)^a^
Other^c^	44.1 (17.8-70.4)	1.50 (0.55-4.13)	44.1 (17.8-70.4)	1.30 (0.48-3.48)	9.2 (0.3-18.1)	1.18 (0.42-3.33)	15.0 (4.8-25.1)	1.34 (0.60-2.98)
Uninsured	33.0 (23.6-42.3)	1.08 (0.6-1.9)	44.2 (34.6-53.9)	1.54 (0.93-2.55)	27.5 (21.3-33.6)	2.46 (1.56-3.88)^a^	42.0 (35.1-48.9)	3.66 (2.43-5.53)^a^
Region								
Northeast	41.1 (28.8-53.5)	[Reference]	44.8 (32.9-56.8)	[Reference]	14.3 (9.2-19.4)	[Reference]	21.5 (15.3-27.6)	[Reference]
Midwest	25.0 (16.8-33.3)	0.51 (0.26-1.01)	31.2 (21.1-41.3)	0.56 (0.28-1.11)	9.8 (6.1-13.4)	0.65 (0.35-1.18)	17.2 (12.7-21.7)	0.67 (0.41-1.09)
South	30.6 (25.3-35.9)	1.68 (0.37-1.25)	35.8 (30.1-41.4)	0.70 (0.38-1.28)	14.7 (11.3-18.1)	0.68 (0.41-1.15)	21.3 (17.2-25.3)	0.67 (0.42-1.05)
West	36.2 (27.3-45.2)	0.76 (0.39-1.47)	41.7 (32.9-50.5)	0.79 (0.42-1.49)	14.1 (9.9-18.3)	0.79 (0.45-1.38)	19.2 (14.7-23.7)	0.71 (0.44-1.15)

Abbreviation: AOR, adjusted odds ratio.

^a^ Statistical significance at *P* < .05.

^b^ Other included the following predefined race categories from the National Health Interview Survey: non-Hispanic American Indian/American Natives only, non-Hispanic American Indian/American Natives and any other group, other single race and multiple races.

^c^ Other was a combined category in the survey that included “other private,” “other public,” and “military” insurance.

## Discussion

A substantial proportion of women who were not vaccinated for HPV never received cervical cancer screening or were not up to date on screening recommendations in 2019. These findings are particularly important in the context of declining cervical cancer screening uptake, recent stabilization in cervical cancer incidence, and the COVID-19 pandemic, which has further exacerbated HPV vaccination and cervical cancer screening rates.^5,6^ For instance, a more than 75% decrease in screening rates occurred among women aged 21 to 29 years during the stay-at-home order in Southern California.^6^ Findings of the present study also suggest that non-Hispanic Asian women, those with educational attainment up to a high school diploma, and those who are uninsured are less likely to undergo or adhere to screening recommendations, implying a need for targeted prevention in these groups. The study limitations include the cross-sectional survey design and the self-reported nature of the data.

Poor cervical cancer screening uptake among US women who are not vaccinated for HPV is a major public health concern. Vigorous efforts are needed to reduce existing screening disparities.
